# Bacteria Hold Their Breath upon Surface Contact as Shown in a Strain of *Escherichia coli*, Using Dispersed Surfaces and Flow Cytometry Analysis

**DOI:** 10.1371/journal.pone.0102049

**Published:** 2014-07-23

**Authors:** Jing Geng, Christophe Beloin, Jean-Marc Ghigo, Nelly Henry

**Affiliations:** 1 Laboratoire Jean Perrin (CNRS FRE 3231), UPMC, Paris, France; 2 Institut Pasteur, Unité de Génétique des Biofilms, Département de Microbiologie, Paris, France; RMIT University, Australia

## Abstract

Bacteria are ubiquitously distributed throughout our planet, mainly in the form of adherent communities in which cells exhibit specific traits. The mechanisms underpinning the physiological shift in surface-attached bacteria are complex, multifactorial and still partially unclear. Here we address the question of the existence of early surface sensing through implementation of a functional response to initial surface contact. For this purpose, we developed a new experimental approach enabling simultaneous monitoring of free-floating, aggregated and adherent cells via the use of dispersed surfaces as adhesive substrates and flow cytometry analysis. With this system, we analyzed, in parallel, the constitutively expressed GFP content of the cells and production of a respiration probe—a fluorescent reduced tetrazolium ion. In an *Escherichia coli* strain constitutively expressing curli, a major *E. coli* adhesin, we found that single cell surface contact induced a decrease in the cell respiration level compared to free-floating single cells present in the same sample. Moreover, we show here that cell surface contact with an artificial surface and with another cell caused reduction in respiration. We confirm the existence of a bacterial cell “sense of touch” ensuring early signalling of surface contact formation through respiration down modulation.

## Introduction

Adherent bacteria profoundly differ from planktonic bacteria in physiology and gene expression. From this collective surface-attached life mode, the bacteria gain significant adaptive advantages and exhibit increased resistance to many biocides [1], [2], [3]. This adhesion-induced physiological shift was suggested very early on by scientists studying bacterial populations in aqueous receptacles [4], [5] and has since been confirmed on the basis of molecular biology data. Recently, abundant information on gene expression and metabolic pathway alterations in established biofilms has emerged due to the increasing spread of molecular genetics [6], [7], [8], [9], [10], [11]. However, the mechanisms of such a transition are not known. The data, obtained on a several hour or day time scale, depict interfering biochemical cascades up- or downregulated in the surface-attached mode of growth compared to the free-floating mode [12]. This reinforces the idea of a surface-attached specific mode of life, but does not enable distinguishing triggering events from further developmental stages that drive biological changes on surfaces. In particular, the respective contributions of the various factors prevailing in biofilms — actual cell surface contact, cell-cell interactions, secreted soluble molecules or extracellular matrix synthesis, together with modifications in the physical and chemical environment due to confinement of cells in a 3D viscoelastic architecture — have not been identified, and their causality remains elusive. In this paper, we focused on the early stage of cell-surface contact formation. Evidence of a direct cell response upon initial adhesion is scarce. Using reporter gene technology and microscope observation in *Pseudomonas aeruginosa* individual cells, Davies and Geesey concluded that attachment of the cell to a glass surface induced *algC* upregulation as early as the first 15 min of contact [13]. In addition, Otto and Silhavy described increased expression of Cpx-regulated genes upon 1 h contact of *Escherichia coli* with artificial surfaces as compared to planktonic cells maintained in suspension; surprisingly, this regulation was observed with stationary phase cells in contact with a hydrophobic surface only [14]. Lately, Li and co-workers showed, in *Caulobacter crescentus*, that formation of physical contact between the bacterium and an artificial surface triggered “just-in-time” adhesin production [15]. These results suggest that bacterial cells possess a ‘tactile’ machinery which signals formation of surface contact. However, the functional responses put forward in these experiments have also been shown to be upregulated in stationary phase cell populations and in bacteria subjected to various external stresses — e.g. nutrient deprivation, medium pH or osmolarity changes — raising the question of the direct relationship of these signals with formation of surface contact.

Here we develop an experimental approach aimed at addressing this question in a configuration which enables simultaneous detection of permanent physical contact and relevant biological activity at the single cell level. The principle of the experiments consisted in using dispersed surfaces in the form of micrometric latex particles as an adhesive substrate brought into contact with GFP-expressing bacterial cells in suspension so as to generate a microsystem in which adherent cells co-exist with single planktonic and aggregated cells. The system can then be characterized using flow cytometry, enabling multi-parametric short-time-scale analysis of the mixture [16]. To detect the impact of initial adhesion on cell metabolic activity, we used a fluorescent marker of bacterial respiration, a tetrazolium ion the fluorescence of which can be directly related to cell metabolic activity [17], [18]. The experiments were performed in an *E. coli* strain constitutively expressing GFP and curli — a surface multimeric protein structure that fosters surface attachment and self-association [19]. The results indicated that bacterial metabolic activity was affected by formation of a single micrometric contact at the cell surface, either with a synthetic surface or with another cell, as early as the first ten minutes of permanent contact formation, suggesting that bacteria have developed an efficient and fast sense of touch. Interestingly, we observed that both cell-cell and cell-synthetic substrate contact triggered a similar metabolic drop. The implications of these findings on the potential existence and possible nature of a bacterial sense of touch will be discussed below. Clarification of these questions will be useful for a better understanding of the physiological shift induced by bacterial cell development on surfaces, a longstanding concern in microbiology.

## Materials and Methods

### Bacterial strain and growth conditions

Constitutive *E. coli* curli producers (MG1655*gfp*_*ompR*234) were in this study obtained by transducing, into *gfp*- tagged MG1655 (MG1655*gfp*), the *ompR*234 mutation that specifies a gain of function allele of *ompR*, a gene encoding an activator of the curli operon [20]. They were grown in lysogeny broth (LB) medium or in M63B1 medium with 0.4% glucose (M63B1Glu) at 37°C in the presence of ampicillin (Amp, 100 µg/ml).

### Chemicals and particles

5-cyano-2,3-ditolyl tetrazolium chloride (CTC), Sytox-red and Syto 59 were purchased from Invitrogen; CTC was from the viability kit (B-34956). Monodispersed polystyrene microparticles of 25 µm and 3 µm diameter — (references 07313-5 and 17134-15, respectively) were purchased from Polysciences, GmbH Europe. Particles were used after extensive washing and re-suspension in M63B1 medium.

### Flow cytometry

FCM analyses were performed using a Becton-Dickinson flow cytometer (Facscalibur) equipped with a 488 nm argon ion laser and 635 nm laser diode. Under 488 nm excitation, GFP emissions were recorded in fluorescence channel FL-1 (band pass 530/30 nm) and CTC in channel FL-3 (long pass >670 nm). Sytox red was excited using the 635 nm laser diode and emission was collected in channel FL-4 (band pass 661/16 nm). Data were analyzed using CellQuest (BD) multivariate analysis and FlowJo software (Tree Star). Fluorescence values were usually obtained from acquisition of 30,000 events, which optimized precision and time resolution. Series of successive acquisitions performed on the same sample exhibited standard deviation below 1% of the mean.

### Confocal microscopy

Image acquisition was performed with a Nikon A1R confocal laser microscope equipped with an argon and NeHe laser using an oil objective lens (60× by 1.4 numerical aperture). Detectors and filter were set for simultaneous monitoring of GFP, CTC and Sytox red. Images were analyzed in imaging software NIS-Elements (Nikon) and Image J.

### Microsystem formation and analysis

We generated a multicellular assemblage microsystem as previously described in detail [16]. Briefly, exponentially growing bacteria in suspension in M63B1Glu of an OD at 600 nm, 1-cm path length about 0.5, were brought into contact with particles at a 1∶200 particle to cell ratio in a round-bottom tube and stirred at room temperature on a soft vortex (1000 rpm.min^−1^). The total volume of this incubator was usually equal to 500 µl. Aliquots of 5 to 20 µl were taken at given incubation times for immediate analysis in FCM. Alternatively, the incubator was used for additional dye staining.

### Fluorescent marker incubation procedures

CTC, Sytox Red and Syto 59 were incubated with cells or cell-particle mixtures at 5 mM, 5 nM and 1 µM final concentration, respectively. Fluorescent dye incubation times were usually equal to 30 min for CTC and 10 min for Sytox red and Syto 59.

## Results

### Combined analysis of CTC reduction and GFP expression provides a single cell respiration index to use in cell assemblages

**Figure 1:**
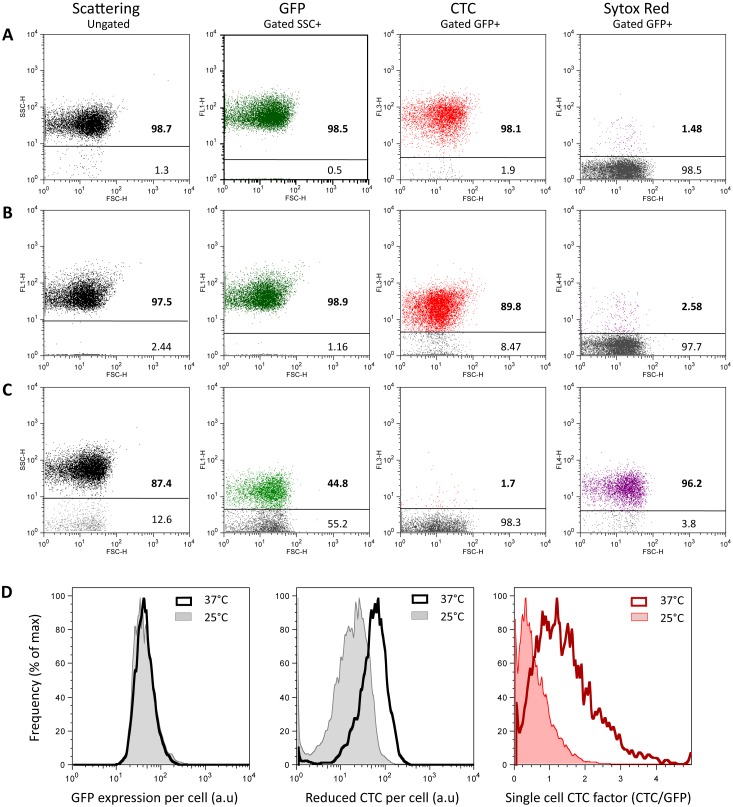
FCM signature of cell respiration down-modulation. Side scattering (SSC, first row) and fluorescence (FL1, second column; FL3, third column; FL4, fourth column) versus forward scattering (FSC) dot plots of bacteria constitutively expressing GFP and labelled with CTC or Sytox red are shown for cells (A) freshly taken from 37°C agitated culture, (B) left to rest at 25°C or (C) fixed in 3.7% formaldehyde. Fluorescence dot plots are from cells exhibiting a positive side scattering signal (>10 a.u). Cell frequencies below and above a fluorescence intensity threshold taken between 4 and 5 a.u. in each channel are indicated on the dot plots (see also Table 1 for fluorescence intensity data). (D) Histograms of fluorescence intensity distribution for GFP expression (left graph), CTC reduction (middle graph) and single cell respiration index (right graph) for cells freshly taken from 37°C agitated culture and left to rest at 25°C.

In order to expose the early bacterial cell response to adhesion, we implemented a strategy consisting of using dispersed surfaces as the adhesive substrate and flow cytometry multiparametric analysis. This approach carried out using GFP-expressing bacteria provides large statistics data sets and time resolution on the order of seconds to determine cell-surface adhesion kinetics. To achieve quantitative monitoring of cell respiration at a single cell level, we first searched for a means of taking advantage of the multiparametric nature of flow cytometry to design a two-color approach to bacterial respiration. For this purpose, we introduced into the experiment a fluorescent marker of bacterial respiration, a tetrazolium ion the fluorescence of which can be directly related to its level of reduction in the electron transfer chain of the bacteria, and thus to the metabolic activity of the cells under study. In its oxidized form, the CTC dye is a non-fluorescent molecule. In contrast, when the compound is reduced via the cell membrane electron transfer chain — in competition with molecular oxygen — it is converted into a water-insoluble product exhibiting characteristic red fluorescence (emission maximum around 602 nm) whose intensity inside the bacterial cell reports the amount of reduced product and thus the cell respiration level, as previously reported [17], [18]. In principle, it is possible, using flow cytometry, to measure the individual cell capacity to reduce CTC and to obtain distribution of this property over a perfectly dispersed cell population. Yet, as soon as cells assemble, the single cell information is lost, except if an internal standard enables counting the number of individuals in the assemblage. For this purpose, we analyzed in parallel the flow cytometry profiles of an exponentially growing *E. coli* cell population constitutively expressing GFP and incubated with CTC. Exponentially growing bacteria cultivated at 37°C under stirring (hereafter referred to as “fresh cells”) were incubated with 5 mM CTC for 30 min just before the flow cytometry test. In parallel, aliquots of the same culture were incubated with Sytox red as a dead cell marker. Incubation time was chosen on the basis of CTC reduction kinetics in cells that indicated that a pseudo-plateau had been reached at that time (see Fig. S1). The same series were prepared with cells taken out of the incubator and left at room temperature for 2 h before the test (bench cells) and with cells previously fixed in 3.7% formaldehyde solution in PBS for 1 h at room temperature (dead cells). FCM dot plots and histograms of GFP, CTC and Sytox red fluorescence intensities collected in their respective emission channels, i.e. FL1, FL3 and FL4, versus forward scattering (FSC), are shown in Fig. 1. Subcellular debris was removed from the analysis by gating the data to an SSC+ region, corresponding to SSC values higher than 10 (a.u. fixed in each channel by the instrument settings chosen at the beginning of the experiment and kept constant for all measurements). Then, we defined for each fluorescence channel a threshold value delimiting positive and negative regions with respect to the considered marker and we determined the corresponding FCM parameters, cell percentage and fluorescence intensity (Table 1). Our results showed that no significant difference in GFP expression or Sytox red labelling was observed between fresh and bench cells. In contrast, both cell distribution between CTC^+^ and CTC^-^ regions and mean fluorescence intensity were noticeably affected (Fig. 1 and Table 1) in bench cells compared with fresh cells, indicating a drop in the reduced CTC production consistent with a cell respiring activity decline caused by a temperature and oxygenation (through sample stirring) decrease. In the extreme situation of the fixed cells, CTC reduction was abolished. Concomitantly, more than 95% of cells exhibited Sytox red labelling, and partial loss of GFP was observed. These results showed that CTC reduction sharply discriminated live from dead cells, as did Sytox red, but also measured graded respiration levels of healthy cells in which GFP content was unchanged on a 2 h time scale. Therefore, GFP intensity could be used as an internal standard to normalize reduced CTC production and define a single-cell respiration index, *f_R_* =  *fl_CTC_/fl_GFP_*. To check that this normalization introduced no spreading or deformation of the value distribution compared to raw *fl_CTC_*, we compared the distributions of *f*_R_ and *fl_CTC_* values normalized to the mean (Fig. 1D). The plot showed superimposable curves endorsing the two-color parameter *f*_R_ as a robust descriptor of respiring activity at a single cell level. In addition, the two-color assessment of bacterial respiration provided a means of deriving a single cell respiration measurement in multiple cell assemblages in which both *fl_GFP_* and *fl_CTC_* could be determined. In these cases, *f*l_GFP_ was not only an internal standard, but also served as a cell counter. An assemblage of *n* cells will have an FL1 fluorescence intensity, 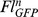 enabling determination of *n* like 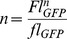, with 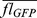 being the mean fluorescence of the co-existing single cells in the same sample. Based on the fact that only CTC but not GFP content was affected by respiring activity changes, single-cell-reduced-CTC content in the *n*-cell assemblage was derived using *n* like 
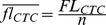
, where *Fl_CTC_* is the FL3 fluorescence of the assemblage, enabling determination of mean single cell respiring activity, and *f*_R_ in the assemblage. This will be used hereafter to determine both surface-attached and self-associated cell respiring activity.

**Table 1:** Scattering and fluorescence FCM characteristics of bacterial cells in high (A) and low (B) metabolic state compared with dead cells (C).

Parameter	Gate + boundary (a.u.)	% Cells in gate+ *		Mean intensity in gate+ (a.u.)**
SSC	10.0	A	98.7	37.8±0.8
		B	97.5	36.5±0.9
		C	87.4	58.7±1.6
GFP (FL1)	4.0	A	98.5	47.8±0.7
		B	98.9	45.5±0.9
		C	44.8	15.1±0.5
CTC (FL3)	5.0	A	98.1	64.3±1.2
		B	89.8	25.1±0.9
		C	1.7	-
Sytox Red (FL4)	4.0	A	1.48	-
		B	2.58	-
		C	96.2	18.1±0.5

*A: Alive cells at 37°C; B: Alive cells at 25°C; C: Dead cells.

**Error is standard deviation over at least three measurements.

### Flow cytometry monitoring of cell-particle microsystem-exposed multiple assemblages

**Figure 2:**
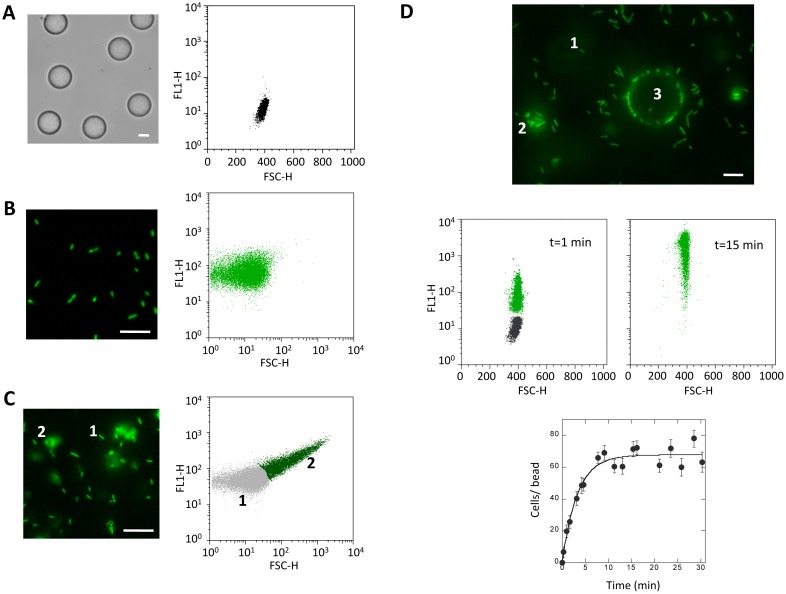
Cell-particle microsystem formation. Microscope image and fluorescence (FL1) versus FSC dot plots of (A) micrometric 25 µm diameter particles alone, (B) single bacteria freshly pipetted to break aggregates, (C) partially aggregated cells as recovered from a usual culture and (D) the cell-particle mixed microsystem comprising single cells (1), aggregated cells (2) and cells adhering to particle surface (3); the two FL1 versus FSC dot plots show the particle signal recorded in the microsystem 1 min and 15 min after bringing cells and particles into contact. Panel D bottom gives cell-particle association kinetics.

To form a cell-particle microsystem in which free-floating, aggregated and adherent cells can be monitored over a short time scale using flow cytometry, we mixed, at a 1∶200 ratio, 25 µm diameter polystyrene particles serving as the adhesion substrate (Fig. 2A) with curli expressing *E. coli* bacteria freshly taken from an exponentially growing culture and pipetted (Fig. 2B). At all desired time points, aliquots were removed from the stirred suspension for immediate analysis by flow cytometry. From the time of the mixture (t = 0), the cells quickly self-assembled and attached to particle surfaces. The microsystem reached its steady state in about 10 min, exhibiting multiple analyzable subgroups of single, surface-attached and self-aggregated cells at various assemblage degrees, as shown on the FL1 (GFP-fluorescence) versus FSC dot plots (Fig. 2C and D). In the example shown in Fig. 2, 98% of the particles carried between 20 to 200 cells and the self-aggregation number for 90% of the aggregates ranged from 2 to 12 cells at t = 15 min.

These results showed that a stable microsystem exhibiting multiple adhesion and self-association degrees could be reproducibly obtained in less than 10 min following mixing of bacteria with particles, with subclasses identifiable and subject to characterization on the basis of their FSC and FL1 parameters in flow cytometry. Unless otherwise stated, the experiments on bacterial respiring activity were performed using this reference microsystem in a steady state.

### Differential respiring activity in single free-floating and assembled cells

**Figure 3:**
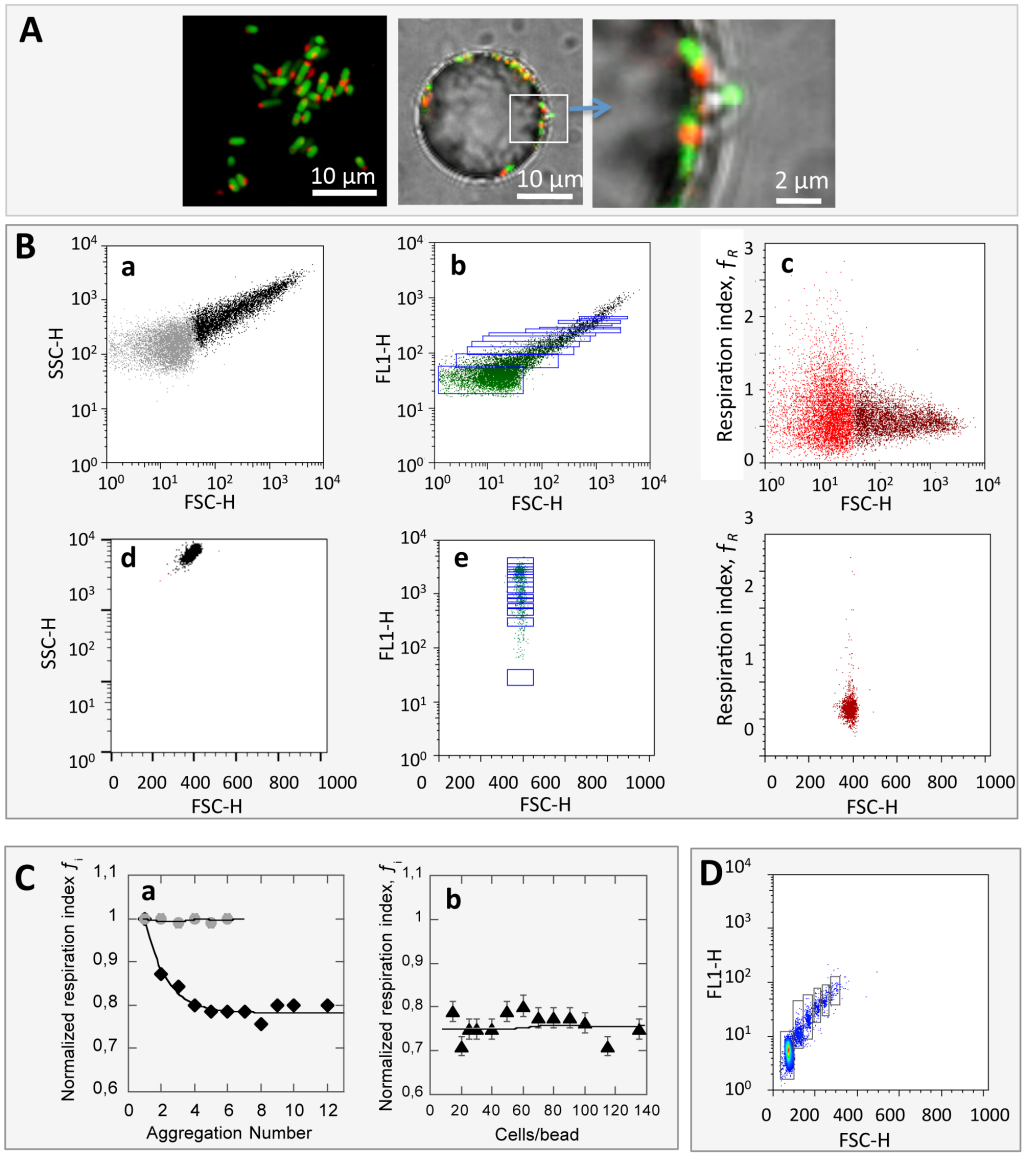
Adherent and aggregated cell respiration down-modulation upon contact. (A) Microscope images of CTC labelled cells in suspension and adhering to the 25 µm diameter particle surface. (B) FCM dot plots: SSC (left panels), FL1 (middle panels) and respiration index *f*_R_ (right panels) versus FSC showing cells (upper row, a to c) — single free-floating cells appear as light gray dots, aggregates as dark dots — and cells adhering to particles (lower row, d to f)); FL1 dot plots (middle panels) reporting GFP expression show cell counting gates, from R_2_ to R_12_ for aggregates and from R_10_ to R_135_ for particles. Gates were defined as explained in the text based on sample single cell mean FL_1_ value. (C) Graphs of normalized respiration index as a function of (a) number of cells (♦) or 2.8 µm diameter beads (•) per aggregate and (b) number of cells per particle. (D) FL1 versus FSC dot plot of 2.8 µm beads used as aggregation controls and population counting gates.

In order to test the respiring activity of the bacterial cells according to their assemblage status, we introduced CTC into the microsystem previously formed by mixing cells and particles for 15 min. Incubation with CTC was completed 30 min before FCM analysis. Reduction of CTC in healthy respiring cells was visible under microscopy by formation of an intracellular red fluorescent speck, as seen in Fig. 3A. Thus GFP and reduced CTC cell content were analyzed in FCM, enabling derivation of the respiration index, using GFP as a cell counter as explained above, for the three subpopulation types present in the microsystem (Fig 3B, panel a-e). In order to compensate for all set-up fluctuations that might interfere with experiments performed some time ago, we used a respiration index normalized to 1 for free-floating cells (
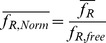
). Then, pooling ten experiments, we found 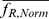 values equal to 0.78±0.04 and 0.75±0.06 for self-aggregated and surface-attached cells, respectively. These results indicated that both surface-attached and self-aggregated cells exhibited decreased respiring activity compared with free-floating cells. To assess the impact of cell assemblage size *n* on respiration down-modulation, we delineated *n* FL1 gates as follows: for all assemblages of *n* cells present in the sample, either forming an aggregate of *n* cells or an assemblage of *n* cells attached to the same particle, an R*_n_* region was defined as including all events having an FL1 value in the interval 
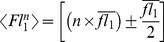
 where 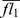 is the mean FL1 value of single free-floating bacteria. By doing this, we obtained adjacent gates with no overlap. For the surface-attached cell population, the particle fluorescence background contribution in the FL1 channel was taken into account to define the gate. With the variation in *f*_R_ as a function of *n*, the assemblage size is shown in Fig. 3C for aggregates and surface-attached cells. The respiration decrease was detected in cell aggregates as small as n = 2 and reached its maximum for aggregates of *n* = 4 cells. This suggested that bacteria can sense even single cell-cell contact. For surface-attached cells, no assemblage size lower than 10 cells per particle with sufficient statistical significance was obtained. We observed that the respiration index decrease experienced by surface-attached cells was not dependent on the number of bound cells, at least between 10 and 100 cells per particle.

To check the robustness of our approach, we used particles having large-spectrum intrinsic fluorescence, enabling a measurement signal in both FL1 and FL3 channels. We induced these particles — 2.8 µm in diameter — to form a small proportion of stable aggregates of a few individuals by centrifugation (Fig. 3D). We then determined an analogous normalized fluorescence index using the same derivations as those applied to cell assemblages. We observed that particle self-association did not affect the normalized fluorescence index (Fig. 3C, panel a). Although the particle number of aggregation did not exceed n = 6, this result showed that an association in itself does not induce a fluorescence shift.

We also challenged our analysis framework by examining the fluorescence of the different bacterial organizations upon labelling with 1 µM Syto 59, a dye which passively penetrates into cells and stains bacterial DNA after insertion between the base pairs without the requirement for metabolic activity. In this case, free-floating and aggregated cells exhibited the same Syto 59 (FL4 signal) to GFP (FL1 signal) fluorescence ratio (Fig. S2), also supporting the metabolic origin of the CTC fluorescence decrease in assembled cells. These results also indicated that the fluorescent dyes equally labelled free-floating and aggregated cells. The same experimental evidence could not be obtained with surface-attached cells, since Syto 59 itself was bound to the particle surface, inducing too high a fluorescence background that blurred the entire analysis. However, results obtained on cell accessibility to fluorescent dyes in aggregates could reasonably be extrapolated to surface-attached cells.

Thus, our results indicate that bacteria detected the formation of cell surface contact and responded to it by metabolism down-modulation, as confirmed by the decrease in respiring activity.

At this stage, we sought to further analyze the respiration activity decrease observed in particle-attached cells so as to determine whether the observed cell response actually resulted from cell-artificial surface anchorage or from cell-cell associations occurring on the particle surface.

### Single cell response to contact

**Figure 4:**
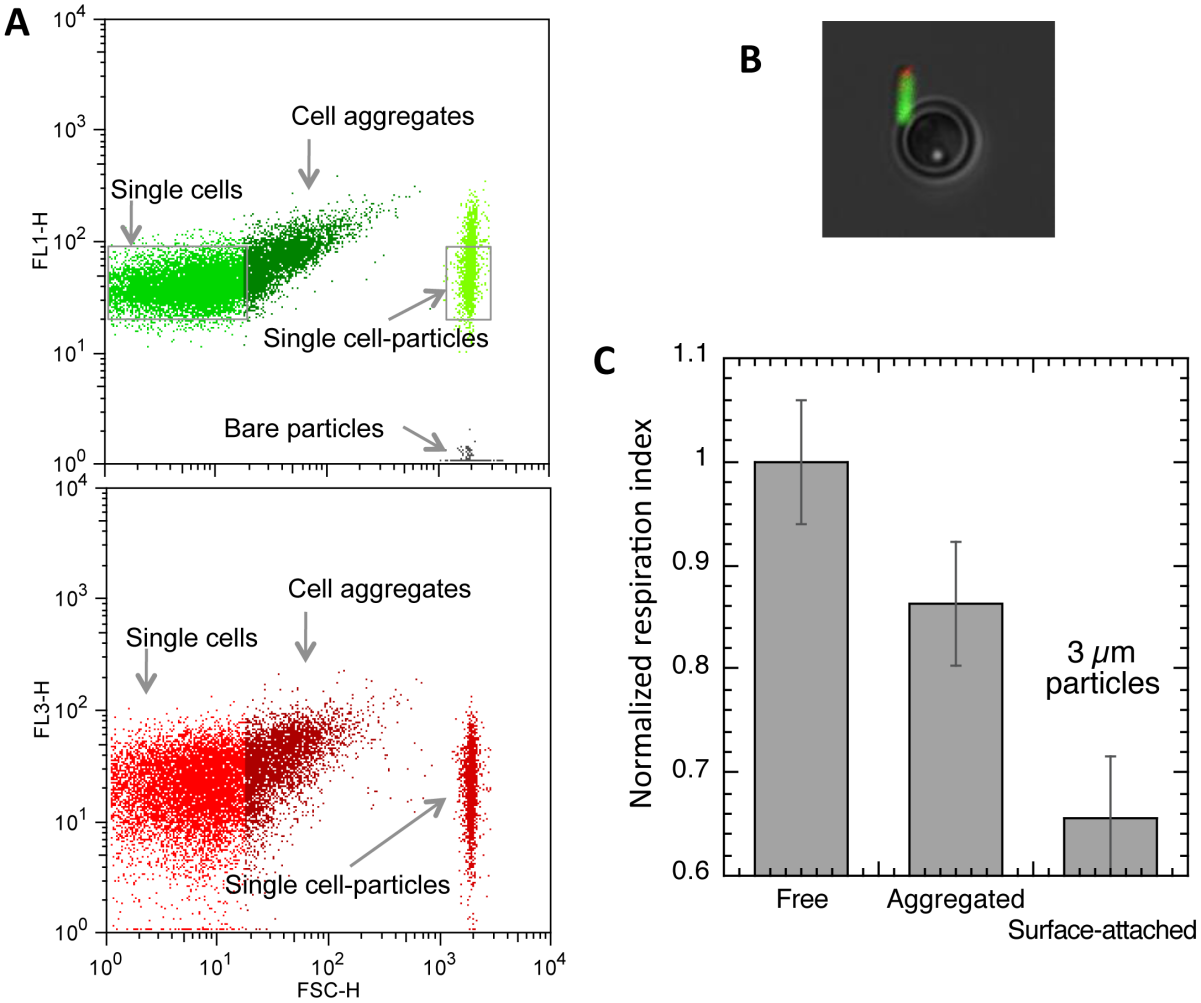
Single cell adhesion assay. (A) FL1 (upper graph) and FL3 versus FSC dot plots of a mixed microsystem of 3 µm particles with single free-floating and aggregated bacteria; microsystem prepared at a cell-to-particle ratio equal to 4 and incubated with 5 mM CTC. (B) Microscope image of a single cell-particle conjugate. (C) Histogram of normalized respiration index of the different cell populations comprised in the same sample, measured 40 min after bringing cells and particles into contact.

In order to discriminate between cell-cell contact on a surface and cell-surface contact, we needed to form a cell-surface assemblage of lower size. For this purpose, we designed an experimental “single-cell adhesion assay” achieved by using smaller-sized particles 3 µm in diameter and decreasing the cell-to-particle ratio to 4, which provided a majority of particles holding one single cell and a small fraction holding none or two cells. Because of the small particle radius, the cells and particles appeared on the same dot plot (Fig. 4). As previously, we defined gates on the basis of FL1 fluorescence so as to delineate single cell events, either free-floating single cells or surface-attached single cells. Again, we derived the normalized respiration index *f*_R,Norm_ for the surface-attached cells and found it equal to 0.7±0.1, which indicated that direct cell-synthetic surface contact also induced a decrease in bacterial respiring activity. The respiration index determined on the self-aggregated cells in the single-cell adhesion assay was found equal to 0.79±0.06, in good agreement with previous results obtained in the presence of the 25 µm-radius particles, as expected, since self-aggregation occurred independently from surface attachment.

### Short-time-scale single cell response to contact

**Figure 5:**
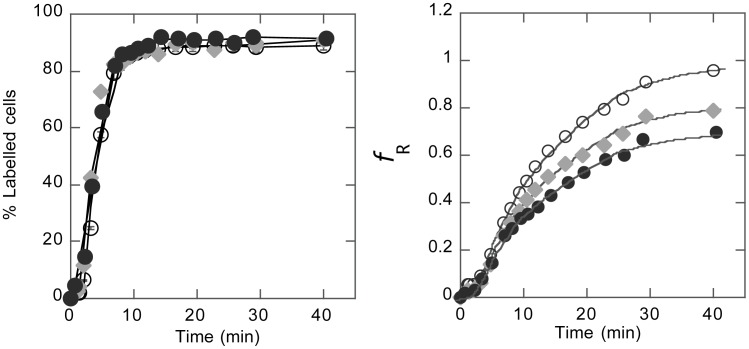
Kinetics of CTC cell influx and reduction. Monitoring (A) CTC positively labelled (FL3>5 a.u.) cell fraction and (B) cell respiration index, ƒ_R_ as a function of CTC incubation time for free-floating (o), aggregated (♦) and adherent (•) cells. CTC reduction reported by FL3 intensity increase in CTC-labelled cells appears to be the limiting (slowest) step in the process.

The results shown above thus indicated that respiring activity decreased upon single cell-cell or cell-substrate contact at the surface of a bacterium after 40 min. This time scale was actually driven by the CTC response time, i.e. reduction rate, and chosen to obtain a sufficiently stable and reduced CTC signal. To evaluate whether a cell response to contact formation occurred earlier than 40 min, we performed kinetic analysis of CTC reduction over the entire period of incubation of the microsystem with CTC. The results displayed in Fig. 5 show that free-floating, aggregated and surface-attached cell respiration indexes diverged from the beginning of incubation. In contrast, the kinetic curves of the reduced CTC labelling frequencies, which report the rate of CTC inflow into the cell independently of the level of reduction, showed no difference between assembled and free-floating cells. Together, these two curve sets suggested that down-modulation of respiring activity had already started after 10 min contact with a facing surface; elsewhere, penetration of the probe did not seem to be affected by contact of the cell either with another cell or with an artificial contact.

## Discussion

Bacteria are primarily found in the form of adherent communities, where they exhibit significant remodeling of their properties compared to planktonic cells. Despite the attention that these changes have garnered, the events determining bacterial physiological shift are still not well understood [12], [21].

In the work reported here, we sought to detect an early cell biological response to formation of cell-surface adhesive contact. Indeed, the phenotypic alterations observed in adherent communities were mainly examined in systems already established for hours or days during which intricate events occurred, including 3D-extracellular matrix formation [22], [23], [24], [25], morphological changes [25] and quorum sensing communication [26], blurring the initial adhesion step and confusing clarification of the various contributions. To gain access to the initial adhesion phase, we implemented a strategy using dispersed surfaces and flow cytometry analysis, which has recently been designed to monitor bacterial short-time-scale adhesion [16]. The technique provides large statistical data sets, time resolution on the order of a few tens of seconds and simultaneous analysis of various-sized objects suspended in the same sample — the reason why flow cytometry has gained significant ground in microbial analyses lately [27], [28]. In this microsystem, we found that *E. coli*, engineered to constitutively produce curli fibers on its surface, reached a steady state exhibiting stable fractions of multidimensional aggregates, planktonic and adherent cells.

To quantitatively picture cell metabolic activity within the first hour of surface colonization, we adapted this strategy to simultaneously follow not only adhesion, but also cell respiration on suspended free-floating, adherent and aggregated cells that co-exist in the microsystem formed when micrometric particles are brought into contact with a bacterial cell culture. This was achieved by introduction of a second fluorescent marker — tetrazolium ion CTC [17], [18]— in addition to GFP constitutively expressed by the cells. In this way, we were able to use GFP expression as an internal standard and cell counter, which enabled unit respiration index determination from CTC and GFP fluorescence intensity measurements.

On the basis of this two-color signature of respiring activity, we demonstrated that CTC reduction monitored at a single cell level in a population of living bacteria could report graded levels of cell respiration within a time scale of 15–20 min.

Implementing this approach in cell-particle mixtures displaying both adherent (particle-attached) and free-floating single cells, we found that the cell-surface association induced a 25 to 30% decrease in the initial cell respiration level.

Interestingly, we also found that cells living in the microsystem in the form of aggregates of various sizes exhibited a similar decrease in respiring activity. Moreover, by examining single-cell/particle associations and low aggregation number (from 2 to 10) cell clusters, we revealed that a single contact was sufficient to initiate this decrease in the respiration level.

These results point to the existence of a bacterial cell “sense of touch”, i.e. the capacity to perceive an external object via formation of a physical contact, implying that bacterial cells have developed molecular pathways to elaborate a biological response to surface physical contact. Such an early single-cell level observation of a substratum-induced response — independently of interfering adhesion secondary events such as confinement, formation of chemical gradients or the onset of extracellular matrix synthesis — had been previously shown by Davies and co-workers in a small number of individuals, based on microscopy monitoring of *algC* gene activation [29]. In comparison, our results rely on data sets of thousands of cells. We show here that surface sensing involves a drop in respiration. Still, we do not know the mechanism behind this process.

Lower and co-workers [30] reported what they called a “tactile” response in *Staphylococcus aureus* after observing — on the basis of atomic force microscopy data — an accumulation of adhesins in the region of cell-substrate contact. Yet, under their experimental conditions, this behavior very likely resulted from thermodynamic equilibration of the free fraction of the binding ligands by diffusion, i.e. a passive process driven by the formation of specific surface receptor/ligand bonds. Our results show a striking parallel between the metabolic down-modulation induced by contact with the synthetic surface of a particle and that induced by cell-cell contact, suggesting that the molecular details of the contact play no determining role in signaling. However, there is no clear-cut conclusion concerning the fundamental difference between cell-artificial surface contact and cell-cell contact in the context of bacterial adhesion. Due to the presence in the cell culture supernatant of a variety of surface active molecules such as proteins and polyelectrolytes, the description of cell/synthetic surface interactions according to a physico-chemical model is hazardous, and the hypothesis of specific interactions between substrate-adsorbed biopolymers and cell surface receptors cannot be ruled out [31]. Again, since experimental data are often the result of biofilms established over several hour or day time scales, at a time when cell-substrate interactions are masked by the predominance of cell-cell interactions, any particular influence of the substrate upon development of the adhesive community is difficult to detect. Here, since we individually and simultaneously monitored each type of contact in the same sample, we were able to demonstrate that formation of cell-cell contact induced a cell response similar to the formation of cell-synthetic surface contact. Recently, based on growth rate, tolerance to antibiotics and extracellular matrix property criteria, Alhede and co-workers [32] pointed out that *Pseudomonas aeruginosa* floating aggregates and surface-attached biofilm shared most phenotypes, suggesting that the substrate per se does not play a determining role in establishment of biofilm properties. These behaviors might, of course, depend on the nature of the adhesive surface, although this point remains difficult to clarify in the context of bacterial adhesion. In our experiments, the polystyrene surface of the particles is expected to be neutral; in fact, it exhibited a slight negative charge (zeta-potential ≈−10 mV) when suspended in cell culture minimal medium (data not shown). In previous work [16], we showed that the particle surface was quickly converted to being negatively charged in the presence of cell culture medium independently of the initial physico-chemical properties of the particles, explaining the similar colonization kinetics observed using different particles. This indicates that the substratum initial properties are often masked by a conditioning film in the presence of bacterial cell culture products.

The similarity of the cell response to a single contact with another cell and with a synthetic surface also enables hypothesizing surface sensing mechanisms driven by physical forces. Indeed, the formation of a contact on the cell surface is likely to induce local mechanical stress of cell surface appendages, which could serve to signal contact formation and trigger the biochemical cascade — as shown for flagella [33]. Other issues such as breaking of cell axial symmetry by formation of surface contact or a reduction in the amount of cell surface exposed to external medium and fluid flows could also be taken into consideration when formulating new working hypotheses for elucidating surface sensing mechanisms.

Our results, which associate respiration reduction with surface sensing, also raise the question of the role of that event in surface contact signaling. Recently, a contact-dependent growth inhibition process involving metabolic down-modulation upon cell-cell contact was discovered in *E. coli* [34], [35]. This phenomenon — mediated by surface-specific protein recognition in an asymmetric inhibitor/target contact involving the immunity protein and toxin translocation across target cell envelopes [36] — is assuredly different from the one observed here. However, it provides an example of a contact-driven bacterial response pathway in which metabolic parameters are reduced in the absence of nutrient deprivation. Moreover, Aoki and co-workers suggested that a respiration decrease could be a general response to formation of physical contact at the cell surface; they proposed that respiration reduction could enable survival under conditions of oxidative stress. Thus far, to the best of our knowledge, no experimental evidence supporting a molecular mechanism linking respiration reduction and contact formation has been published.

Conversely, other authors detected metabolism stimulation events upon surface adhesion [37]. However, their experiments were performed under 12 h starvation conditions, thus significantly differing from our own experimental conditions.

Here we provide new tools for investigating in more detail this metabolic shift induced by surface contact at the single-cell level. Our approach enables discriminating between sensing mediated by soluble secreted factors and direct physical cell surface contact by monitoring planktonic and attached cells exposed in parallel to the same medium. This is a key issue in the better understanding of the mechanisms driving adherent cell transition towards an altered physiological state. Our results indicate that the reduction in the cell respiration level participates in early signaling triggered upon cell aggregation or settlement on an artificial substrate.

These results raise many questions for further investigation. What is the impact of this reduction on overall cell functioning, and, for instance, on the cell division rate? Is this a way for cells growing in the form of attached communities to achieve greater tolerance to oxidative stress or to various antimicrobials? What are the molecular bases for this contact-dependent behavior? The design of specific short-lifetime genetic reporters, to be monitored in our multiparametric dispersed surface approach, will undoubtedly help to answer these questions.

## Supporting Information

Figure S1 **CTC reduction kinetics.** (A) Fraction of CTC-positively-labelled (FL3>5 a.u.) cells and (B) cell respiration index, *f*_R_ as a function of CTC incubation time. Incubation with 5 mM CTC at 37°C under stirring.
(TIF)

Figure S2 **A neutral labelling index in free-floating and aggregated cells.** Syto 59 (FL4 signal) to GFP (FL1 signal) fluorescence ratio calculated from fluorescence dot plots for free-floating (light grey triangle) and aggregated (black diamond) cells. Incubation with 1 µM Syto59 at 37°C under stirring.
(TIF)
