# Supplementary figures and images for: Bacteria Hold Their Breath upon Surface Contact as Shown in a Strain of Escherichia coli, Using Dispersed Surfaces and Flow Cytometry Analysis
Source: PLoS One. 2014 Jul 23;9(7):e102049. doi: 10.1371/journal.pone.0102049 (PMC4108326; doi:10.1371/journal.pone.0102049)

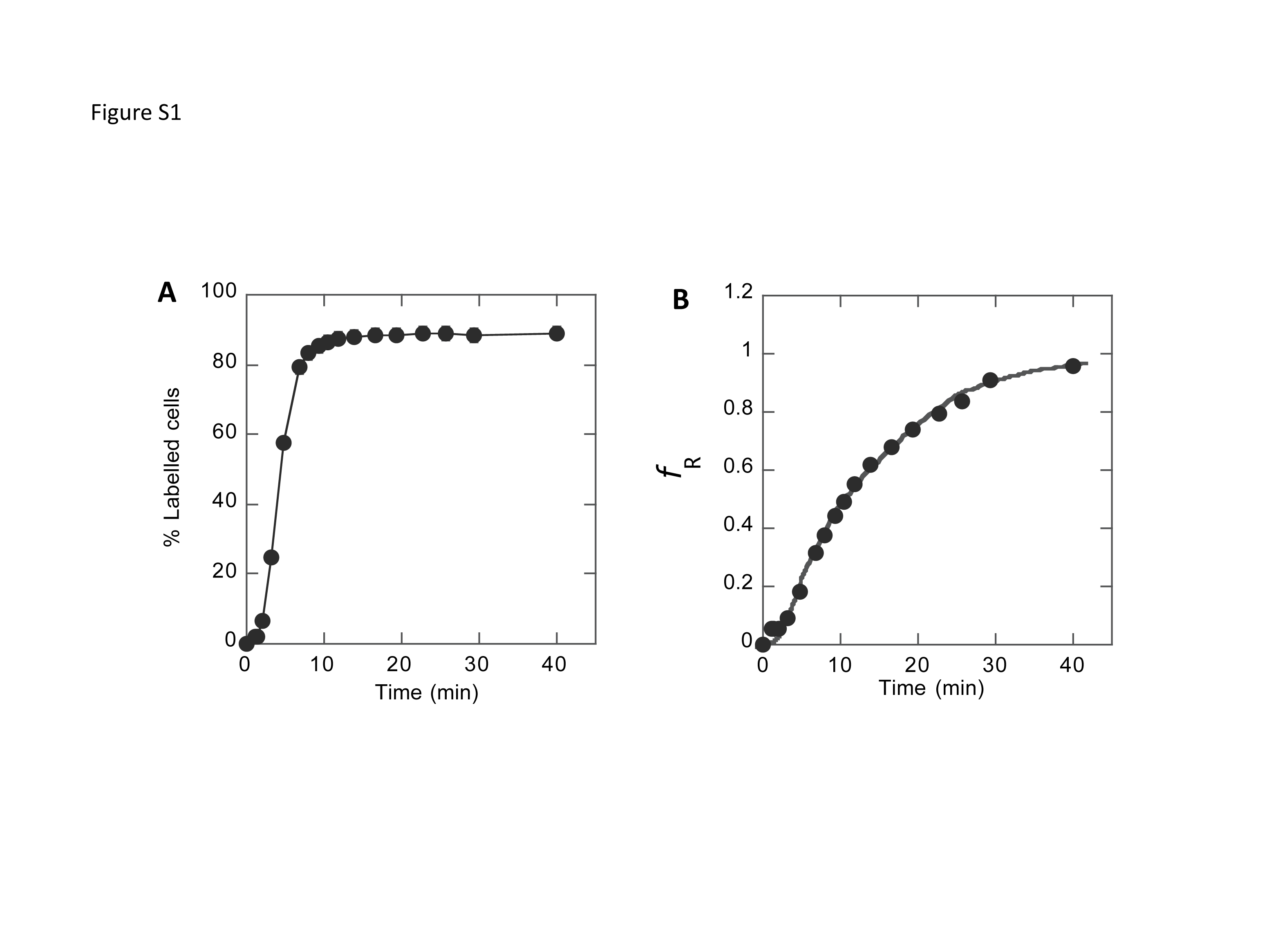

Supplement: Figure S1 — CTC reduction kinetics. (A) Fraction of CTC-positively-labelled (FL3>5 a.u.) cells and (B) cell respiration index, f R as a function of CTC incubation time. Incubation with 5 mM CTC at 37°C under stirring. (TIF) [file pone.0102049.s001.tif]

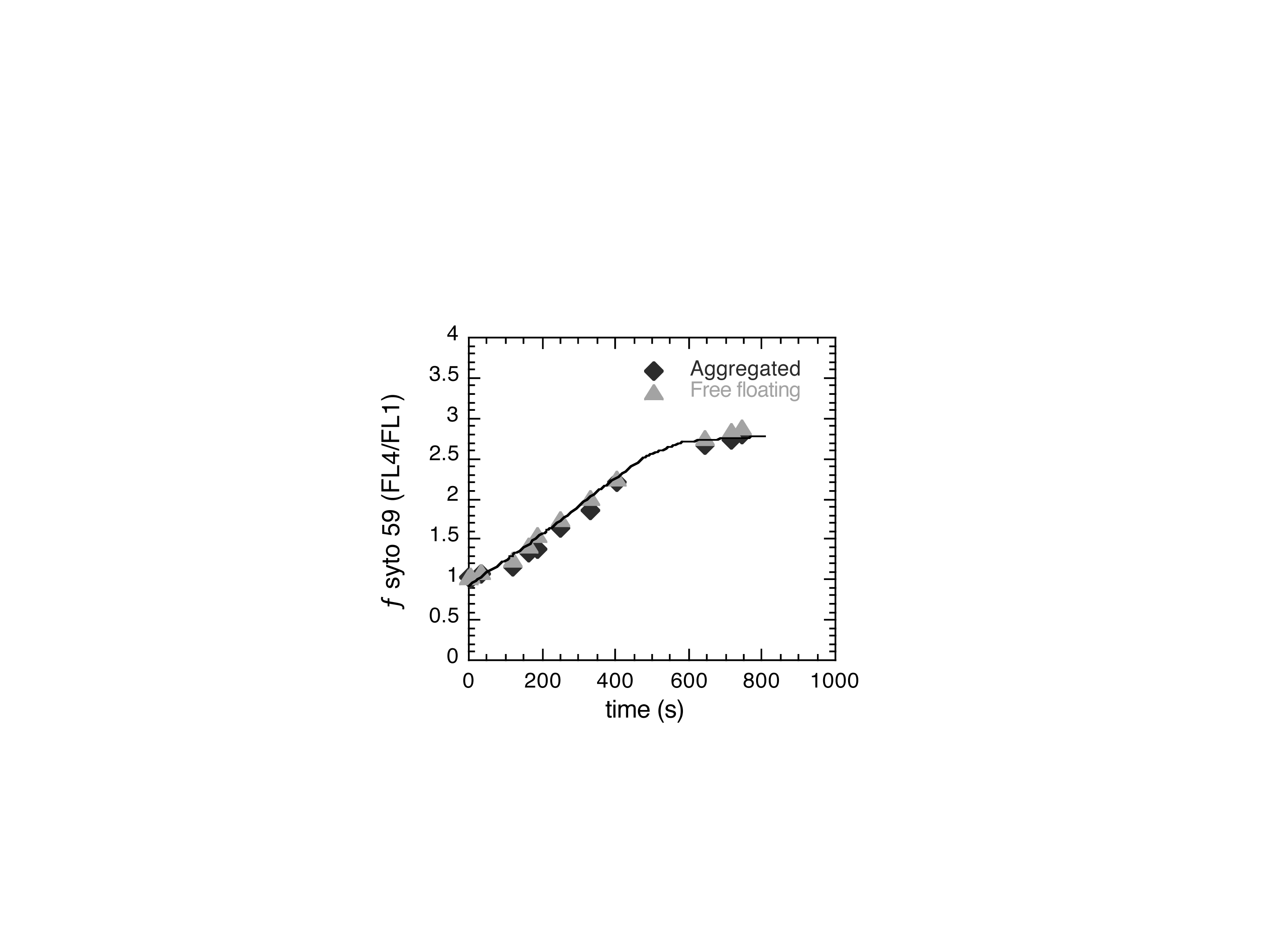

Supplement: Figure S2 — A neutral labelling index in free-floating and aggregated cells. Syto 59 (FL4 signal) to GFP (FL1 signal) fluorescence ratio calculated from fluorescence dot plots for free-floating (light grey triangle) and aggregated (black diamond) cells. Incubation with 1 µM Syto59 at 37°C under stirring. (TIF) [file pone.0102049.s002.tif]
